# Transcriptional Control of TSPAN32 in T-ALL Reveals Interplay Between TAL1 and NOTCH1

**DOI:** 10.3390/biomedicines13092090

**Published:** 2025-08-27

**Authors:** Grazia Scuderi, Antonio Arcidiacono, Eugenio Cavalli, Maria Sofia Basile, Antonella Nardo, Ferdinando Nicoletti, Paolo Fagone

**Affiliations:** 1 Department of Biomedical and Biotechnological Sciences, University of Catania, 95123 Catania, Italy; graziascuderi@hotmail.it (G.S.); a.arcidiacono@unict.it (A.A.); eugeniocavalli9@hotmail.it (E.C.); ferdinic@unict.it (F.N.); 2 Department of Medicine and Surgery, “Kore” University of Enna, 94100 Enna, Italy; mariasofia.basile@unikore.it; 3 Division of Haematology, A.O.U. Policlinico “G. Rodolico-S. Marco”, 95123 Catania, Italy; antonella.nardo5@gmail.com

**Keywords:** tetraspanins, TSPAN32, TSSC6, Phemx, leukemia

## Abstract

**Background:** T-cell acute lymphoblastic leukemia (T-ALL) is an aggressive malignancy of immature T cells, driven by dysregulated transcriptional networks and oncogenic signaling pathways. Here, we present the first comprehensive analysis of the expression and regulation of TSPAN32, a tetraspanin implicated in lymphocyte homeostasis, in T-ALL. **Methods**: Transcriptomic data from the Leukemia MILE study (GSE13159) were analyzed to assess TSPAN32 expression across leukemic subtypes. Gene Set Enrichment Analysis (GSEA) was performed to explore biological pathways associated with TSPAN32-correlated genes. For mechanistic validation, HPB-ALL cells were used as a model, with NOTCH signaling inhibited by γ-secretase inhibitor (GSI) treatment and TAL1–LMO1 overexpression induced through doxycycline-inducible lentiviral vectors. Gene expression changes were quantified by RT-qPCR. **Results**: TSPAN32 was frequently downregulated in T-ALL compared to healthy bone marrow, although expression was retained in a subset of cases. GSEA revealed that TSPAN32-correlated genes were inversely associated with cell cycle–related programs, consistent with its established role as a negative regulator of T cell proliferation. Mechanistically, TAL1–LMO1 overexpression strongly induced TSPAN32, while GSI-mediated NOTCH inhibition partially reactivated its expression. Interestingly, GSI treatment also increased TAL1 levels despite downregulating LMO1. Conversely, TAL1–LMO1 overexpression suppressed NOTCH1 and NOTCH3, highlighting a reciprocal regulatory interplay between NOTCH and TAL1/LMO1 oncogenic circuits that shapes TSPAN32 expression dynamics in T-ALL. **Conclusions**: This study identifies TSPAN32 as a novel transcriptional target under the influence of key leukemogenic pathways and suggests its potential role as a modulator of leukemic T cell proliferation, with implications for therapeutic strategies targeting TAL1 and NOTCH signaling.

## 1. Introduction

T-cell acute lymphoblastic leukemia (T-ALL) is an aggressive hematologic malignancy that arises from the malignant transformation of T-cell precursors in the thymus. It is characterized by uncontrolled cellular proliferation, impaired differentiation, and widespread genomic alterations affecting critical regulatory pathways [1]. T-ALL accounts for approximately 15% of pediatric and 25% of adult acute lymphoblastic leukemia cases. While treatment outcomes have significantly improved in children—with long-term survival rates ranging between 70% and 90%—the prognosis in adults remains less favorable, with five-year survival rates hovering around 50%. This discrepancy underscores the need for improved understanding of disease mechanisms and the identification of new molecular targets for therapeutic intervention [1].

Recent advances in transcriptomics and next-generation sequencing have provided insight into the molecular landscape of T-ALL. These studies have highlighted the pivotal role of transcription factor dysregulation in driving leukemogenesis. Distinct molecular subtypes of T-ALL have been classified based on the ectopic expression of lineage-defining transcription factors, including TAL1, TLX1, TLX3, HOXA, LMO2/LYL1, and NKX2-1. Among these, the TAL1 subgroup is the most prevalent, accounting for 40–60% of cases. TAL1 (T-cell acute lymphocytic leukemia protein 1) functions as a basic helix-loop-helix (bHLH) transcription factor and exerts its oncogenic activity by forming multi-protein complexes with E-proteins (such as TCF3/E2A and TCF12/HEB), the LIM-domain proteins LMO1 or LMO2, and the transcription factors GATA3, RUNX1, and MYB [2,3].

These TAL1-centered transcriptional complexes orchestrate the expression of genes that promote leukemic proliferation and block differentiation [4]. TAL1 not only regulates downstream oncogenic targets such as ALDH1A2 and ARID5B but also participates in the autoregulation of its own transcriptional network, forming a tightly interconnected core regulatory circuit. This regulatory network maintains the leukemic state by reinforcing the expression of TAL1 itself and its cofactors, thereby sustaining the oncogenic transcriptional program [4]. Concurrently, activation of the NOTCH1 signaling pathway represents another hallmark of T-ALL, observed in roughly half of all patients. Aberrant NOTCH1 activity, often due to activating mutations in NOTCH1 or inactivating mutations in the ubiquitin ligase FBXW7, leads to the constitutive expression of MYC and other pro-proliferative genes. Furthermore, there is compelling evidence that NOTCH1 cooperates with TAL1 in driving leukemogenesis, particularly in TAL1-positive T-ALL, by synergistically activating a subset of shared target genes [5].

While the roles of TAL1 and NOTCH1 in T-ALL are well documented, little is known about how these oncogenic programs interact with genes that regulate immune cell quiescence and homeostasis. TSPAN32 is a member of the tetraspanin family [6,7,8], which has been previously implicated in the maintenance of T-cell quiescence and the negative regulation of proliferation [9,10]. However, its expression pattern and regulatory mechanisms in T-ALL have not been systematically explored. In this study, we aimed to fill this gap by investigating the transcriptional regulation of TSPAN32 in T-ALL and its relationship to key oncogenic drivers such as TAL1 and NOTCH1. We sought to determine whether TSPAN32 is differentially expressed in leukemic versus healthy hematopoietic cells, to identify potential transcriptional networks that modulate its expression, and to assess its potential role as a tumor suppressor or modulatory factor within the leukemic transcriptional landscape. Our findings provide new insight into the integration of oncogenic and quiescence-associated transcriptional programs in T-ALL, and may open new avenues for therapeutic intervention aimed at restoring differentiation and halting leukemic proliferation.

## 2. Materials and Methods

### 2.1. Patient Transcriptomic Data Analysis

To investigate TSPAN32 expression across hematologic malignancies, we analyzed publicly available transcriptomic data from the Leukemia MILE study (GSE13159) [11,12], which includes Affymetrix Human Genome U133 Plus 2.0 microarray profiles of over 2000 patient samples covering major leukemia subtypes and healthy controls. We selected data corresponding to T-ALL (*n* = 174), B-cell ALL subtypes with t(12;21) translocation (*n* = 58), mature B-ALL with [t(8;14) (*n* = 13), chronic lymphocytic leukemia (CLL) (*n* = 448), and healthy bone marrow (n = 74). Expression values were log_2_-transformed and normalized using the RMA algorithm. TSPAN32 expression was extracted and compared across diagnostic groups to determine differential expression patterns relative to normal bone marrow.

### 2.2. Gene Co-Expression and Enrichment Analysis

To assess the functional landscape associated with TSPAN32 in T-ALL, we performed correlation analysis using expression data from T-ALL patient samples within the GSE13159 dataset. Pearson correlation coefficients were calculated for all genes in relation to TSPAN32, and the top positively correlated genes were selected for further investigation. Hierarchical clustering was performed using Euclidean distance and complete linkage to visualize co-expression relationships in heatmap format. The biologically relevant functions of TSPAN32-associated genes were explored using Gene Set Enrichment Analysis (GSEA), employing the MSigDB Hallmark (https://www.gsea-msigdb.org/gsea/index.jsp) (accessed on 5 May 2025) and GO Biological Process collections as the reference background, implemented in the web-based application WebGestalt (https://www.webgestalt.org/#) (accessed on 5 May 2025) [13]. Enrichment significance was assessed using the normalized enrichment score (NES) and false discovery rate (FDR) q-values.

### 2.3. Cell Culture and Experimental Treatments

The human T-cell acute lymphoblastic leukemia (T-ALL) cell line, HPB-ALL, were used to study the regulation of TSPAN32 under NOTCH and TAL1 signaling modulation. Cells were cultured in RPMI-1640 medium supplemented with 10% fetal bovine serum (FBS), 1% penicillin-streptomycin, and 2 mM L-glutamine, under standard conditions (37 °C, 5% CO_2_). Cells were seeded at a density of 5 × 10^5^ cells/mL and treated for 12 h with the γ-Secretase inhibitor (GSI) XXI (compound E) was used at 1.0 µM final concentration to pharmacologically inhibit NOTCH receptor cleavage and activation. DMSO at 0.01% final concentration served as vehicle control.

Full-length TAL1 and LMO1 cDNA sequences were cloned into the doxycycline-inducible lentiviral vector pCW57.1 (Addgene) to enable controlled overexpression in HPB-ALL cells. Lentiviral transduction was performed, and 48 h post-infection, drug selection was applied for 36 h. Following selection, cells were treated with doxycycline to induce expression of the transgenes.

#### 2.3.1. RNA Extraction and cDNA Synthesis

Following treatment, total RNA was extracted using the RNeasy Mini Kit (Qiagen) according to the manufacturer’s instructions, including on-column DNase digestion to eliminate genomic DNA contamination. RNA quantity and integrity were assessed using a NanoDrop 2000 spectrophotometer and agarose gel electrophoresis. One microgram of total RNA was reverse transcribed using the iScript™ cDNA Synthesis Kit (Bio-Rad) in a 20 μL reaction volume, following the manufacturer’s protocol. The resulting cDNA was diluted 1:5 in nuclease-free water prior to quantitative PCR analysis.

#### 2.3.2. Quantitative Real-Time PCR (qRT-PCR)

Gene expression was quantified by real-time PCR using iTaq™ Universal SYBR^®^ Green Supermix (Bio-Rad) on a CFX96 Touch™ Real-Time PCR Detection System (Bio-Rad). Each 20 μL PCR reaction contained 2 μL of diluted cDNA, 10 μL of SYBR Green Supermix, and 300 nM each of forward and reverse primers. Primers were designed to span exon-exon junctions when possible, and specificity was verified by melting curve analysis and agarose gel electrophoresis. The following genes were analyzed: ARID5B, ALDH1A2, IL7R, BCL11B, HEY1, HEY2, HES1, FBXW7, DTX1, NRAP, NOTCH1, NOTCH2, NOTCH3, NOTCH4, TAL1, LMO1, MYB, GATA3 and TSPAN32. GAPDH was used as the internal normalization control, as its expression has been demonstrated to be stable in leukemic models, including T-ALL, across both cell lines and patient-derived samples [14,15]. Primer sequences were obtained from the PrimerBank database (https://pga.mgh.harvard.edu/primerbank/) (accessed on 5 May 2025). All reactions were performed in technical triplicates.

### 2.4. Statistical Analysis

Statistical comparisons of gene expression across patient groups were performed using one-way ANOVA followed by FDR post hoc test for multiple comparisons. Correlation coefficients were computed using Spearman’s method, and significance thresholds were set at FDR < 0.05 and r ≥ |0.4|. GSEA significance was determined using a false discovery rate (FDR) q < 0.05 as the cutoff for enriched pathways. Relative gene expression was calculated using the 2^–ΔΔCt^ method, with expression normalized to GAPDH and reported as %variation relative to the mock- or vehicle-treated control group. A *p*-value < 0.05 was considered statistically significant. All analyses were conducted using R (v4.3.0) and GraphPad Prism (v9).

## 3. Results

### 3.1. TSPAN32 Expression Patterns in T-ALL and Hematologic Malignancies

**Figure 1:**
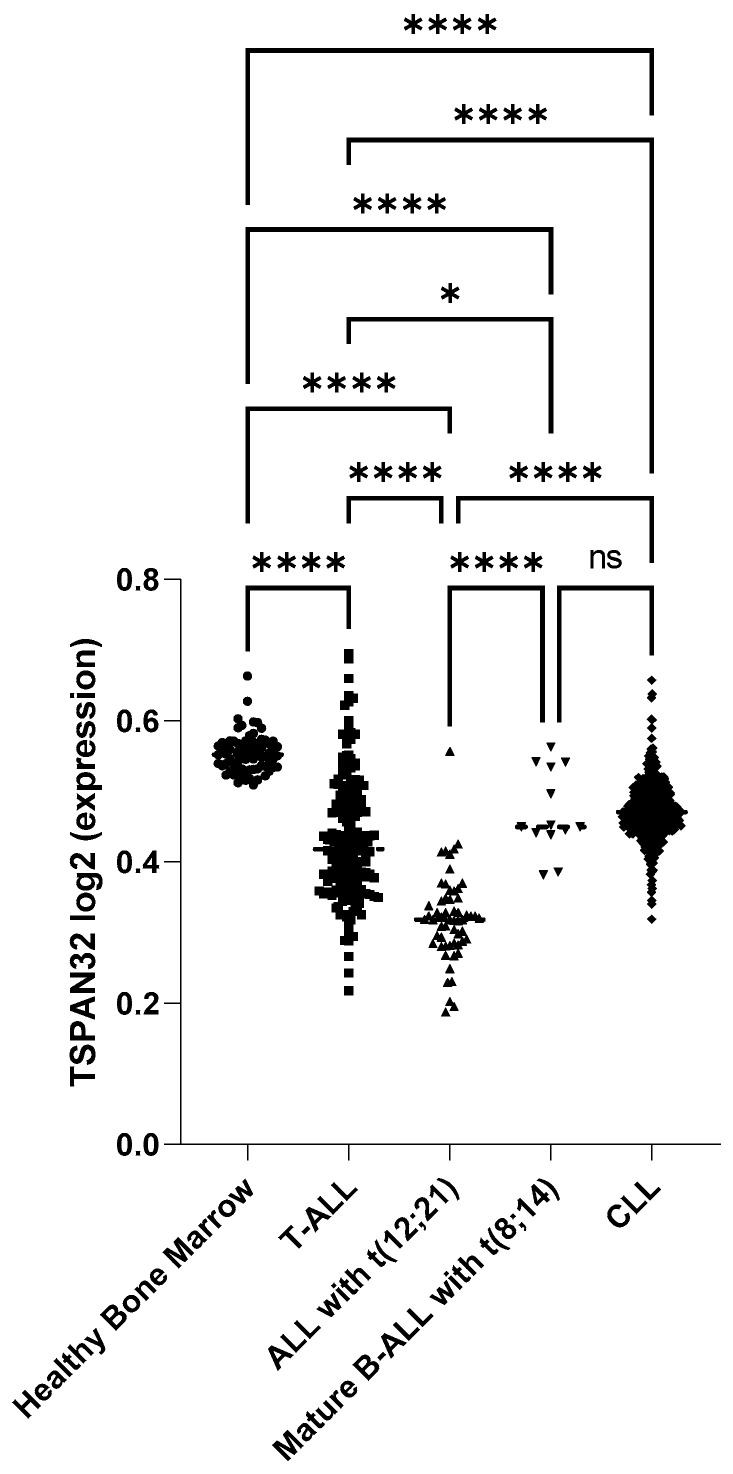
TSPAN32 is significantly downregulated in T-ALL compared to healthy bone. Log_2_-transformed expression levels of TSPAN32 were analyzed in microarray data from the MILE study (GSE13159), comparing healthy bone marrow (n = 74), T-ALL (n = 174), ALL with t(12;21) (*n* = 58), mature B-ALL with t(8;14) (*n* = 13), and CLL (*n* = 448). * *p* < 0.05; **** *p* < 0.0001; ns: not significant by ANOVA followed by FDR post hoc test.

To assess TSPAN32 expression across hematologic malignancies, we analyzed microarray data from the Leukemia MILE study (GSE13159), which includes transcriptomic profiles of T-ALL, B-cell acute lymphoblastic leukemia (ALL with t(12;21) and mature B-ALL with t(8;14)), chronic lymphocytic leukemia (CLL), and healthy bone marrow. TSPAN32 expression was significantly reduced in all leukemic subtypes when compared to healthy bone marrow. In T-ALL samples, the downregulation of TSPAN32 was statistically significant (*p* < 0.001), although a subset of cases displayed expression levels overlapping with those of healthy donors. Similar patterns of reduced expression were observed in B-ALL subtypes and CLL, suggesting widespread suppression of TSPAN32 across lymphoid malignancies (Figure 1).

### 3.2. Co-Expression Landscape and Pathway Enrichment of TSPAN32-Associated Genes

We next investigated the co-expression profile of TSPAN32 in T-ALL using Pearson correlation analysis of transcriptomic data derived from the MILE dataset. Genes significantly correlated with TSPAN32 were identified and subjected to hierarchical clustering.

**Figure 2:**
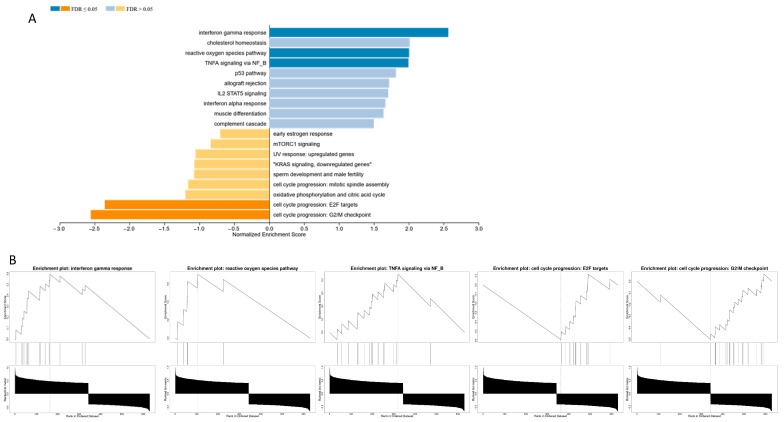
Genes co-expressed with TSPAN32 in T-ALL are enriched in immune activation and cell cycle pathways. (**A**) Gene Set Enrichment Analysis (GSEA) of the top correlated genes. (**B**) Significant pathways identified by GSEA, FDR < 0.05.

Gene Set Enrichment Analysis (GSEA) was performed using the MSigDB Hallmark and GO Biological Process gene sets to identify biological pathways associated with genes correlated with TSPAN32. Enrichment analysis revealed statistically significant associations with multiple immune-related and stress response pathways. Specifically, the positively correlated gene set showed significant enrichment for TNF-alpha signaling via NF-κB (normalized enrichment score [NES] = 2.13, FDR q < 0.01), interferon gamma response (NES = 2.01, FDR q < 0.01), and reactive oxygen species pathway (NES = 1.92, FDR q < 0.05) (Figure 2B). Additional enrichment was observed in pathways related to regulation of leukocyte activation, apoptosis, and inflammatory response. In contrast, negatively correlated genes showed enrichment for cell cycle progression and mitotic pathways, suggesting an inverse relationship between TSPAN32 expression and proliferative gene programs.

### 3.3. Regulation of TSPAN32 by TAL1, LMO1, and NOTCH1 Signaling in T-ALL Cells

**Figure 3:**
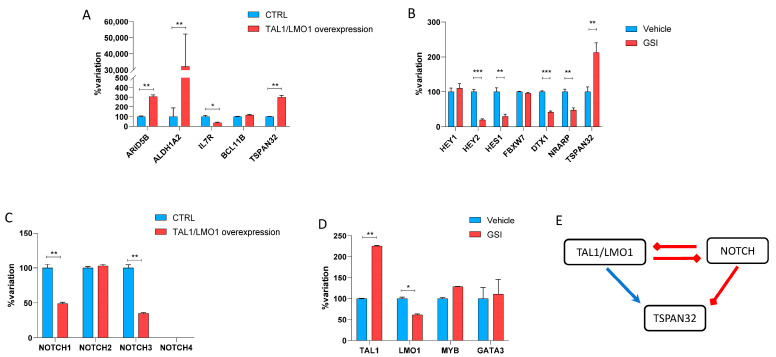
Transcriptional regulation of TSPAN32 in T-ALL. (**A**) Bar graph showing the relative gene expression variation (% of baseline) for TSPAN32 and TAL1/LMO1 targets in HPB-ALL cells upon TAL1/LMO1 overexpression. (**B**) Bar graph showing the relative gene expression variation (% of baseline) for TSPAN32 and Notch signaling targets in HPB-ALL cells upon GSI treatment. (**C**) Bar graph showing the relative gene expression variation (% of baseline) for NOTCH1-4 in HPB-ALL cells upon TAL1/LMO1 overexpression. (**D**) Bar graph showing the relative gene expression variation (% of baseline) for TAL1/LMO1 signaling genes in HPB-ALL cells upon GSI treatment. Expression levels were quantified by qRT-PCR and normalized to GAPDH. (**E**) Proposed regulatory circuit of TSPAN32 expression in T-ALL. * *p* < 0.05; ** *p* < 0.01; *** *p* < 0.001 by ANOVA followed by FDR post hoc test.

TAL1 is a master oncogenic driver in a major subset of T-ALL, and LMO1 is a frequent cofactor in TAL1-driven leukemias. Previous studies indicate that TAL1/LMO1 complexes regulate transcriptional programs critical for leukemogenesis, making them biologically relevant regulators to test for their impact on TSPAN32 expression (reviewed in [4]). Also, a crucial role has been established for NOTCH in T-ALL [5]. To investigate the regulatory mechanisms controlling TSPAN32 expression in T-ALL, we used HPB-ALL cells and modulated the TAL1/LMO1 and Notch signaling pathways. Under basal conditions, TSPAN32 mRNA expression was low or barely detectable. Co-expression of TAL1 with LMO1 resulted in TSPAN32 upregulation, with mRNA levels reaching approximately 3-fold over baseline (*p* < 0.01) (Figure 3A). The induction of TSPAN32 in response to TAL1/LMO1 overexpression occurred in parallel with increased expression of known TAL1 target genes, including ALDH1A2 and ARID5B.

To assess the role of NOTCH signaling in TSPAN32 regulation, cells were treated with the γ-secretase inhibitor (GSI) XXI (compound E) at a final concentration of 1.0 µM for 12 h. TSPAN32 expression increased by approximately 2-fold following GSI treatment compared to DMSO-treated controls (*p* < 0.05), indicating partial reactivation of transcription upon NOTCH inhibition (Figure 3B). Concurrently, expression of canonical NOTCH target genes, such as HES1, HEY1, and DTX1, was significantly reduced in GSI-treated cells, confirming effective pathway inhibition.

We also evaluated the effect of TAL1/LMO1 overexpression on NOTCH receptor expression. TAL1/LMO1 overexpression resulted in a modest but statistically significant downregulation of NOTCH1 and NOTCH3 mRNA (Figure 3C), whereas NOTCH2 levels remained unchanged and NOTCH4 was not detectable under any condition. Treatment with GSI significantly increased the expression levels of TAL1, while a modest but significant downregulation of LMO1 was observed (Figure 3D).

Overall, these findings delineate a transcriptional circuit in which TSPAN32 is upregulated by TAL1/LMO1 overexpression, but suppressed under active NOTCH signaling. The proposed model, illustrated in Figure 3E, supports a dual regulatory framework wherein TAL1/LMO1 complexes activate TSPAN32 expression while NOTCH signaling contributes to its repression.

## 4. Discussion

In the present study, for the first time, we have investigated the expression pattern of TSPAN32 in T-ALL, identifying a consistent trend of downregulation in the majority of analyzed cases. This finding aligns with previously reported data in chronic myeloid leukemia (CML) [16] and our observations in Burkitt lymphoma [17], further supporting a potential tumor-suppressive role for TSPAN32 across distinct hematologic malignancies. Importantly, our results indicate that TSPAN32 downregulation is not restricted to T-ALL, as reduced expression was also observed in B-ALL and chronic lymphocytic leukemia (CLL), highlighting a broader pattern of suppression that transcends specific lymphoid lineages.

Nonetheless, it is critical to note that not all leukemia samples exhibited lower TSPAN32 expression relative to healthy controls, suggesting that TSPAN32 repression is neither universal nor attributable to a single pathogenic mechanism. This variability highlights the complexity of transcriptional regulation in leukemia and the possibility that TSPAN32 expression may be preserved in certain molecular subtypes or disease contexts. Unfortunately, the MILE dataset used for the present study does not provide sufficient molecular annotation to determine whether this reflects specific oncogenic subtypes. Future studies integrating detailed genomic and transcriptomic profiling will be necessary to clarify whether TSPAN32 regulation is subtype-dependent.

Supporting its hypothesized role in regulating lymphocyte proliferation [8,9,10], TSPAN32 expression showed a robust inverse correlation with genes involved in cell cycle progression using GSEA. These associations are in line with previous reports, particularly the work by Tarrant et al. [8], which characterized TSPAN32 as a negative regulator of T-cell proliferation, suggesting its involvement in maintaining immune cell quiescence.

These observations led us to investigate upstream regulators that may modulate TSPAN32 expression in the leukemic context. We first focused on the transcription factor TAL1, whose dysregulation is a hallmark of approximately 40–60 percent of human T-ALL cases and often arises through chromosomal translocations (e.g., t (1;14) (p32;q11)) that lead to aberrant expression of TAL1 in T-cell progenitors [18]. TAL1 functions as a class II basic helix–loop–helix (bHLH) factor that heterodimerizes with the class I E-proteins E2A (TCF3) and HEB (TCF12) to regulate transcriptional programs governing hematopoietic differentiation [4,19]. Consistent with this hypothesis, ChIP-seq datasets from ENCODE demonstrate occupancy of TAL1 at proximal regulatory elements upstream of TSPAN32 in K562 and GM12878 cells, and co-localization with H3K27ac marks has been observed in public ChIP-seq tracks (ENCODE track genome.ucsc.edu), supporting a role for active chromatin and TAL1-driven transcriptional activation at this locus. Given that TAL1-LMO1 complexes have been previously shown to recruit transcriptional coactivators, such as EP300 [20], it is plausible that TAL1/LMO1 repositions chromatin architecture at the TSPAN32 locus and thereby converts a silenced promoter into an acetylated, transcriptionally competent state.

We next examined the role of NOTCH1 signaling in the regulation of TSPAN32. Activating mutations in NOTCH1 [21], most frequently within the heterodimerization and PEST domains, occur in more than 60 percent of T-ALL cases and drive oncogenesis by promoting proliferation, inhibiting apoptosis, and blocking differentiation. Inhibition of NOTCH1 via γ-secretase inhibitor (GSI) treatment resulted in a moderate yet reproducible increase in TSPAN32 expression, suggesting that active NOTCH1 signaling contributes to sustaining TSPAN32 repression. Supporting the hypothesis of direct transcriptional regulation, JASPAR in silico analysis identified putative RBPJ binding motifs within the TSPAN32 promoter region, consistent with canonical NOTCH-RBPJ-mediated transcriptional control. Interestingly, GSI treatment also led to a significant increase in TAL1 expression, despite concurrently downregulating LMO1. Conversely, overexpression of TAL1 and LMO1 resulted in the downregulation of both NOTCH1 and NOTCH3, highlighting a complex regulatory interplay between the NOTCH and TAL1/LMO1 oncogenic circuits that may influence TSPAN32 expression dynamics in T-ALL.

Taken together, the data support a regulatory network in which TSPAN32 expression in T-ALL is governed by the interplay of TAL1-centered transcriptional complexes, active NOTCH1 signaling, and the epigenetic environment at its promoter. Specifically, we propose that TAL1/LMO1 complexes act as transcriptional activators of TSPAN32, independently of their repressive effect on NOTCH1/3, whereas active NOTCH signaling directly suppresses TSPAN32 expression. In this framework, TSPAN32 expression reflects the relative dominance of TAL1-mediated activation versus NOTCH-driven repression, thus explaining why inhibition of NOTCH by GSI relieves repression and increases TSPAN32, and why TAL1/LMO1 overexpression similarly promotes its transcription. Additional epigenetic mechanisms—such as DNA methylation of promoter CpGs and Polycomb-mediated H3K27 trimethylation—may further regulate TSPAN32 transcriptional levels.

Despite the consistency of our findings with existing literature and bioinformatic predictions, several limitations must be acknowledged. First and foremost, most of our conclusions derive from in vitro experiments in established T-ALL cells, which—while valuable for mechanistic dissection—may not fully recapitulate the complex microenvironmental and clonal heterogeneity of primary leukemia specimens. Indeed, T-ALL is marked by distinct genetic subtypes, including those driven by TAL1 translocations, TLX1/TLX3 rearrangements, and HOXA overexpression, each with unique transcriptional landscapes and epigenetic profiles [22]. Our observation that TSPAN32 downregulation is widespread across T-ALL, B-ALL, and CLL suggests a broadly conserved oncogenic program; however, the lack of TSPAN32 repression in a subset of patients implies that specific genetic lesions—or compensation by alternative transcriptional networks—may preserve TSPAN32 expression in certain contexts. For instance, TLX3-rearranged T-ALL cases [23], may not enforce TSPAN32 silencing to the same extent, potentially explaining why some samples maintain normal transcript levels. Comprehensive profiling of TSPAN32 expression across genetically annotated patient cohorts will be necessary to delineate the precise relationship between leukemia subtype, underlying mutations (e.g., NOTCH1, FBXW7, PHF6, HOXA cluster abnormalities), and TSPAN32 regulation. Also, we have to point out that a limitation of the present work is that mechanistic validation experiments were performed in a single T-ALL cell line (HPB-ALL). While this line is a well-established TAL1-negative, GSI-sensitive model [24], thus providing a relevant cellular context to investigate the regulatory interplay between NOTCH signaling, TAL1 complexes, and TSPAN32, reliance on one cellular context may not fully capture the heterogeneity of T-ALL. Additional validation across multiple T-ALL models would strengthen the generalizability of our findings. Moreover, patient-derived xenograft models or primary T-ALL samples would provide further confirmation of the regulatory interplay between TSPAN32, TAL1, and NOTCH1 in a more physiological setting. These aspects represent important directions for future investigations.

Second, while we have shown that overexpression of TAL1/LMO1 and GSI treatment can induce TSPAN32, we have not yet performed targeted chromatin immunoprecipitation experiments to confirm direct binding of these factors to TSPAN32 regulatory regions in T-ALL cells. Publicly available ChIP-seq data from ENCODE provide evidence of TAL1 occupancy at regions proximal to TSPAN32 in non-T-ALL cell lines; however, cell-type–specific binding events in T-ALL remain to be validated. Indeed, ENCODE data indicate that TAL1 ChIP-seq peaks in K562 cells overlap with E-box motifs upstream of the TSPAN32 transcription start site (genome.ucsc.edu), but whether TAL1 and its cofactors similarly occupy this locus in T-ALL remains unknown. To address this gap, future studies should employ ChIP-seq or ChIP-qPCR using antibodies against TAL1, LMO1/2, HES1, and associated histone marks (H3K27ac, H3K27me3) in leukemic cell lines and primary blasts.

Third, although our GSEA suggests that TSPAN32 expression is inversely correlated with cell cycle gene expression (with significant enrichment of the “cell cycle progression: E2F targets” and “cell cycle progression: G2/M checkpoint” biological processes)—an observation that aligns with literature indicating that TSPAN32 promotes quiescence in immune cells [8,25]—definitive functional validation of TSPAN32 as a tumor suppressor in T-cell leukemia is still required. Specifically, forced overexpression of TSPAN32 in T-ALL cell lines should be evaluated for effects on proliferation, cell cycle progression, apoptosis, and clonogenic potential. Additionally, transcriptome profiling following TSPAN32 restoration could reveal downstream targets involved in cell cycle arrest, metabolic reprogramming, or immune signaling, thereby clarifying the mechanistic basis of its potential tumor-suppressive function. Nevertheless, the negative regulatory function of TSPAN32 in lymphocytes has been widely documented. Tarrant et al. (2002) [8] first reported that TSPAN32 acts as a suppressor of T-cell activation and proliferation, while Gartland et al. (2010) [25] confirmed that its loss enhances T-cell proliferative responses. More recently, Qiu et al. (2023) [16] provided evidence linking TSPAN32 with reduced proliferative programs in human lymphocytes, and Scuderi et al. (2025) [17,26] showed its inverse association with oncogenic transcriptional networks in hematologic malignancies.

Fourth, our study has not yet explored other mechanisms of regulation that may influence TSPAN32 expression. Post-transcriptional mechanisms—such as microRNA–mediated silencing—could also contribute to low TSPAN32 levels in leukemia. Epigenetic mechanisms beyond histone modifications, such as higher-order chromatin loops and topologically associating domains (TADs), may also restrict access of transcriptional machinery to the TSPAN32 promoter.

Finally, although we have identified TAL1/LMO1, and NOTCH1 as key nodes in the regulatory network controlling TSPAN32 expression, it is likely that additional transcription factors and cofactors contribute to fine-tuning its transcriptional output. For example, genome-wide analyses from the ChEA database indicate that transcriptional regulators such as SP1, CTCF, and MYC can bind to TSPAN32 regulatory regions in various cell types, and these interactions may be co-opted in leukemia to reinforce silencing or enable activation under specific conditions. Thus, future investigations should consider the dynamic interplay among multiple transcriptional regulators, chromatin modifiers, and signaling pathways to fully characterize the TSPAN32 regulatory network.

In conclusion, our work provides novel insights into the transcriptional and epigenetic mechanisms governing TSPAN32 expression in T-ALL. We demonstrate that TSPAN32 is broadly downregulated in these leukemias and inversely correlates with cell cycle gene expression, supporting a model in which TSPAN32 acts as a quiescence-promoting factor whose silencing facilitates oncogenic proliferation. Mechanistically, we uncover a dual regulatory circuit in which overexpression of TAL1 and LMO1, can relieve TSPAN32 silencing and drive its transcription, whereas active NOTCH1 signaling contributes to its repression. Because TAL1 and NOTCH1 mutations define the genomic landscape of a large fraction of T-ALL cases, our findings integrate TSPAN32 into the core transcriptional framework of this disease. Moreover, confirming the direct binding of TAL1 isoforms and NOTCH1 effectors to TSPAN32 regulatory elements, as well as determining the functional impact of TSPAN32 reactivation on leukemia cell behavior, will be critical next steps. Ultimately, a deeper understanding of TSPAN32 regulation may uncover novel therapeutic opportunities to restore quiescence programs in malignant lymphoid cells, potentially improving outcomes for patients with T-ALL. Importantly, our findings raise the possibility that TSPAN32 modulation could synergize with existing therapeutic approaches. For example, GSIs which target NOTCH signaling, partially reactivated TSPAN32 expression in our model, suggesting that therapeutic strategies capable of restoring TSPAN32 could enhance the efficacy of NOTCH blockade. Given the limited success of GSIs as monotherapies due to toxicity and resistance [27,28,29,30,31], combining them with approaches that upregulate tumor-suppressive genes like TSPAN32 may provide a rational path toward improved treatment strategies. Future studies integrating pharmacologic modulation of TSPAN32 with current therapeutic regimens, including NOTCH inhibitors or TAL1/LMO1-targeted strategies, will therefore be critical to explore the translational significance of our findings.

## Data Availability

Data are publicly available, or they are available upon reasonable request to the corresponding author.

## References

[1] Buckley M., Yeung D.T., White D.L., Eadie L.N. (2025). T-Cell Acute Lymphoblastic Leukaemia: Subtype Prevalence, Clinical Outcome, and Emerging Targeted Treatments. Leukemia.

[2] Kanagal-Shamanna R., Weinberg O.K., Bueso-Ramos C.E. (2025). Genomics of T-Lymphoblastic Leukemia/Lymphoma: 2023 SH/EAHP Workshop Proceedings. Am. J. Clin. Pathol..

[3] Xu J., Teachey D.T. (2025). Genomic Classification of T-Cell Acute Lymphoblastic Leukemia: Current Paradigms and Future Biomarkers. J. Natl. Compr. Cancer Netw..

[4] Vagapova E.R., Spirin P.V., Lebedev T.D., Prassolov V.S. (2018). The Role of TAL1 in Hematopoiesis and Leukemogenesis. Acta Naturae.

[5] Lu J., Xue X., Wang H., Hao Y., Yang Q. (2025). Notch1 Activation and Inhibition in T-Cell Acute Lymphoblastic Leukemia Subtypes. Exp. Hematol..

[6] Nicholson R.H., Pantano S., Eliason J.F., Galy A., Weiler S., Kaplan J., Hughes M.R., Ko M.S. (2000). Phemx, a Novel Mouse Gene Expressed in Hematopoietic Cells Maps to the Imprinted Cluster on Distal Chromosome 7. Genomics.

[7] Robb L., Tarrant J., Groom J., Ibrahim M., Li R., Borobakas B., Wright M.D. (2001). Molecular Characterisation of Mouse and Human TSSC6: Evidence That TSSC6 Is a Genuine Member of the Tetraspanin Superfamily and Is Expressed Specifically in Haematopoietic Organs. Biochim. Biophys. Acta.

[8] Tarrant J.M., Groom J., Metcalf D., Li R., Borobokas B., Wright M.D., Tarlinton D., Robb L. (2002). The Absence of Tssc6, a Member of the Tetraspanin Superfamily, Does Not Affect Lymphoid Development but Enhances In Vitro T-Cell Proliferative Responses. Mol. Cell. Biol..

[9] Basile M.S., Mazzon E., Mangano K., Pennisi M., Petralia M.C., Lombardo S.D., Nicoletti F., Fagone P., Cavalli E. (2020). Impaired Expression of Tetraspanin 32 (TSPAN32) in Memory T Cells of Patients with Multiple Sclerosis. Brain Sci..

[10] Lombardo S.D., Mazzon E., Basile M.S., Campo G., Corsico F., Presti M., Bramanti P., Mangano K., Petralia M.C., Nicoletti F. (2019). Modulation of Tetraspanin 32 (TSPAN32) Expression in T Cell-Mediated Immune Responses and in Multiple Sclerosis. Int. J. Mol. Sci..

[11] Kohlmann A., Kipps T.J., Rassenti L.Z., Downing J.R., Shurtleff S.A., Mills K.I., Gilkes A.F., Hofmann W.-K., Basso G., Dell’orto M.C. (2008). An International Standardization Programme towards the Application of Gene Expression Profiling in Routine Leukaemia Diagnostics: The Microarray Innovations in Leukemia Study Prephase. Br. J. Haematol..

[12] Haferlach T., Kohlmann A., Wieczorek L., Basso G., Kronnie G.T., Béné M.-C., De Vos J., Hernández J.M., Hofmann W.-K., Mills K.I. (2010). Clinical Utility of Microarray-Based Gene Expression Profiling in the Diagnosis and Subclassification of Leukemia: Report from the International Microarray Innovations in Leukemia Study Group. J. Clin. Oncol..

[13] Elizarraras J.M., Liao Y., Shi Z., Zhu Q., Pico A.R., Zhang B. (2024). WebGestalt 2024: Faster Gene Set Analysis and New Support for Metabolomics and Multi-Omics. Nucleic Acids Res..

[14] Gupta D.G., Varma N., Sreedharanunni S., Abdulkadir S.A., Naseem S., Sachdeva M.U.S., Binota J., Bose P., Malhotra P., Khadwal A. (2023). Evaluation of Adverse Prognostic Gene Alterations & MRD Positivity in BCR::ABL1-like B-Lineage Acute Lymphoblastic Leukaemia Patients, in a Resource-Constrained Setting. Br. J. Cancer.

[15] Gupta D.G., Varma N., Kumar A., Naseem S., Sachdeva M.U.S., Bose P., Binota J., Gupta M., Sonam P., Rana P. (2022). Identification and Validation of Suitable Housekeeping Genes for Gene Expression Studies in BCR-ABL1 Positive B-Lineage Acute Lymphoblastic Leukemia. Mol. Biol. Rep..

[16] Qiu Q., Sun Y., Yang L., Li Q., Feng Y., Li M., Yin Y., Zheng L., Li N., Qiu H. (2023). TSPAN32 Suppresses Chronic Myeloid Leukemia Pathogenesis and Progression by Stabilizing PTEN. Signal Transduct. Target. Ther..

[17] Scuderi G., Mangano K., Leone G.M., Fagone P., Nicoletti F. (2025). TCF3 and ID3 Regulate TSPAN32 Expression in Burkitt Lymphoma. Scand. J. Immunol..

[18] Dai Y.-T., Zhang F., Fang H., Li J.-F., Lu G., Jiang L., Chen B., Mao D.-D., Liu Y.-F., Wang J. (2022). Transcriptome-Wide Subtyping of Pediatric and Adult T Cell Acute Lymphoblastic Leukemia in an International Study of 707 Cases. Proc. Natl. Acad. Sci. USA.

[19] Smith C., Charbonnier G., Simonin M., Balducci E., Steimle T., Andrieu G.P., Cieslak A., Courgeon M., LeLorc’h M., Mayakonda A. (2025). Towards Methylation-Based Redefinition of TAL1 Positive T-Cell Acute Lymphoblastic Leukaemia (T-ALL). Leukemia.

[20] Sanda T., Lawton L.N., Barrasa M.I., Fan Z.P., Kohlhammer H., Gutierrez A., Ma W., Tatarek J., Ahn Y., Kelliher M.A. (2012). Core Transcriptional Regulatory Circuit Controlled by the TAL1 Complex in Human T Cell Acute Lymphoblastic Leukemia. Cancer Cell.

[21] de Smith A.J. (2025). Toward Equitable Risk Classification for All Patients with T-ALL. Blood Cancer Discov..

[22] Iacobucci I., Mullighan C.G. (2017). Genetic Basis of Acute Lymphoblastic Leukemia. J. Clin. Oncol..

[23] E S., Jelloul F.Z., Nahmod K.A., Short N., Leventaki V., Jia F., Xu J., Loghavi S., Wang W., Jabbour L.E. (2025). Acute Lymphoblastic Leukaemia with T- and B-Lineage Defining Markers. Pathology.

[24] Choi A., Illendula A., Pulikkan J.A., Roderick J.E., Tesell J., Yu J., Hermance N., Zhu L.J., Castilla L.H., Bushweller J.H. (2017). RUNX1 Is Required for Oncogenic Myb and Myc Enhancer Activity in T-Cell Acute Lymphoblastic Leukemia. Blood.

[25] Gartlan K.H., Belz G.T., Tarrant J.M., Minigo G., Katsara M., Sheng K.-C., Sofi M., van Spriel A.B., Apostolopoulos V., Plebanski M. (2010). A Complementary Role for the Tetraspanins CD37 and Tssc6 in Cellular Immunity. J. Immunol..

[26] Scuderi G., Mangano K., Petralia M.C., Basile M.S., Di Raimondo F., Fagone P., Nicoletti F. (2025). Comprehensive Analysis of TSPAN32 Regulatory Networks and Their Role in Immune Cell Biology. Biomolecules.

[27] Bertulfo K., Perez-Duran P., Miller H., Ma C., Ambesi-Impiombato A., Samon J., Mackey A., Lin W.-H.W., Ferrando A.A., Palomero T. (2025). Therapeutic Targeting of the NOTCH1 and Neddylation Pathways in T Cell Acute Lymphoblastic Leukemia. Proc. Natl. Acad. Sci. USA.

[28] Lehal R., Zaric J., Vigolo M., Urech C., Frismantas V., Zangger N., Cao L., Berger A., Chicote I., Loubéry S. (2020). Pharmacological Disruption of the Notch Transcription Factor Complex. Proc. Natl. Acad. Sci. USA.

[29] Kaushik B., Pal D., Saha S. (2021). Gamma Secretase Inhibitor: Therapeutic Target via NOTCH Signaling in T Cell Acute Lymphoblastic Leukemia. Curr. Drug Targets.

[30] Franciosa G., Smits J.G.A., Minuzzo S., Martinez-Val A., Indraccolo S., Olsen J.V. (2021). Proteomics of Resistance to Notch1 Inhibition in Acute Lymphoblastic Leukemia Reveals Targetable Kinase Signatures. Nat. Commun..

[31] Suzuki S., Hourai S., Uozumi K., Uchida Y., Yoshimitsu M., Miho H., Arima N., Ueno S.-I., Ishitsuka K. (2022). Gamma-Secretase Inhibitor Does Not Induce Cytotoxicity in Adult T-Cell Leukemia Cell Lines despite NOTCH1 Expression. BMC Cancer.

